# The serum levels of angiopoietin‐like protein 3 and 4 in type 2 diabetic patients with and without metabolic syndrome compared to the control group

**DOI:** 10.1002/edm2.466

**Published:** 2023-12-23

**Authors:** Sharabeh Hezarkhani, Aytekin Hajighaderi, Sara Hosseinzadeh, Naser Behnampour, Gholamreza Veghari, Farshid Fathabadi, Zahra Hesari, Hamid Reza Joshaghani

**Affiliations:** ^1^ Metabolic Disorders Research Center Golestan University of Medical Sciences Gorgan Iran; ^2^ Laboratory Sciences Research Center Golestan University of Medical Sciences Gorgan Iran; ^3^ Department of Biostatistics, Faculty of Health Golestan University of Medical Sciences Gorgan Iran; ^4^ Ischemic Disorders Research Center Golestan University of Medical Sciences Gorgan Iran

**Keywords:** ANGPTL 3, ANGPTL 4, diabetes, metabolic syndrome

## Abstract

**Introduction:**

ANGPTLs (Angiopoietin‐like proteins) 3 and 4 play an important role in the development of type 2 diabetes. These glycoproteins affect the modulation of glucose and lipid metabolism. They inhibit lipoprotein lipase (LPL) activity and provoke lipolysis. This study was aimed to investigate the protein levels of ANGPTL3 and 4 in the serum of type 2 diabetic patients with metabolic syndrome in comparison to the type 2 diabetic patients without metabolic syndrome and the control group.

**Methods:**

Three groups of individuals were included in this study; Group I: 47 patients with type 2 diabetes and metabolic syndrome; Group II: 25 patients with type 2 diabetes without metabolic syndrome; Group III: 40 non‐diabetic healthy people without metabolic syndrome as a control group. After collection of 5 mL fasting blood samples, serum concentrations of fasting blood sugar (FBS), cholesterol (Chol), triglyceride (TG), HDL‐C (High‐density lipoprotein‐Cholesterol) and LDL‐C (Low‐density lipoprotein‐Cholesterol) were measured by the enzymatic method; blood pressure (BP), height and weight with stadiometers; and ANGPTL3 and 4 by the enzyme‐linked immunosorbent assay (ELISA).

**Results:**

The serum levels of ANGPTL3 was significantly different among our three groups (*p* = .000). In patients with type 2 diabetes and metabolic syndrome (Group I), ANGPTL3 and 4 levels were lower than the control group.

The serum levels of the parameters evaluated in this study (except HDL‐C) was lower in the group II in comparison with the group I, and this difference was significant for TG, Chol, BP and BMI between these two groups. Also, our results revealed that there was a negative correlation between FBS, TG, Chol, LDL‐C and BMI with ANGPTL3 and 4. While, there was a significant positive correlation between ANGPTL4 and ANGPTL3.

**Conclusion:**

Altogether, our findings suggest that the decreased levels of ANGPTL3 and 4 may be a causative factor for type 2 diabetes.

## Graphical abstract

ANGPTL protein play an important role in the development of type 2 diabetes. These glycoproteins affect the modulation of glucose and lipid metabolism. They inhibit lipoprotein lipase activity and provoke lipolysis.

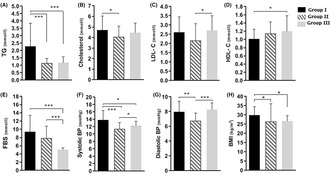

## 1 INTRODUCTION

Metabolic syndrome is a set of health disorders that puts the person at an elevated risk for cardiovascular diseases and type 2 diabetes. Its symptoms range from mild dyslipidemia and impaired fasting glucose levels to fully developed type 2 diabetes.^1^, ^2^, ^3^ The aetiology of the disease is not completely clear yet and it is believed that three categories of the etiological factors including insulin resistance, disorders of adipose tissue and a collection of other independent factors are implicated in its onset.^4^

Most of the patients with metabolic syndrome have no symptoms of diabetes before the development of classic diabetes.^4^, ^5^ Therefore, it is valuable to look for methods and factors to predict metabolic syndrome.

The angiopoietin‐like protein (ANGPTL) family is a family with eight members (ANGPTL1 through to ANGPTL8) which are structurally identical to the angiopoietins.^6^ These proteins are secretory glycoproteins and show pro‐angiogenic effects.^7^ Some members of the ANGPTL family have an effective role in obesity, insulin resistance, and diabetes. The most studied glycoproteins of this family are ANGPTL3 and 4, on the basis of their impact on the regulation of lipoprotein lipase (LPL) activity.^8^, ^9^ These proteins are mainly secreted by the liver into the systemic circulation and affect the function of the numerous tissues such as white adipose tissue.^10^ Although, there is a structural similarity of 31% between ANGPTL3 and 4 their biological function varies depending on the underlying conditions.^11^, ^12^, ^13^

ANGPTL 3 is a key regulator of lipoprotein metabolism and mainly inhibits LPL activity in the fed state. Its gene expression is transcriptionally modulated by the liver X receptor (LXR). LXR is an important lipid sensing transcription factor that acts as a sensor of cholesterol metabolism and lipid biosynthesis.^14^, ^15^ Also, studies have shown that cholesterol‐feeding in mice results in the upregulation of ANGPTL3.^14^

ANGPTL4 gene expression is regulated by the peroxisome proliferator‐activated receptors that are implicated in the modulation of lipid metabolism and insulin sensitivity. ANGPTL4 inhibits LPL enzyme in both fasted and fed states.^6^, ^16^

Although, both ANGPTL3 and ANGPTL4 inhibit LPL activity and induce lipolysis differences in the regulation of their gene expression may confer specific functions of each in lipoprotein metabolism.^6^, ^17^

Due to the increasing rate of diabetes and the importance of ANGPTL3 and ANGPTL4 in the pathogenesis of diabetes through possible association with metabolic syndrome, it seems there is a relationship between these glycoproteins with metabolic syndrome especially in patients with diabetes.^17^, ^18^ Therefore, our study was designed to investigate the serum levels of ANGPTL 3 and 4 in type 2 diabetic patients with metabolic syndrome in comparison with the type 2 diabetic patients without metabolic syndrome and the control group.

## 2 MATERIALS AND METHODS

### 2.1 Subjects

This study included three groups of volunteer participants. 72 individuals with type 2 diabetes (23 males and 49 females) of mean age 53.3 ± 12.21 years (range 24–86), as study population referred to the medical centers and were grouped according to the National Cholesterol Education Program's Adult Treatment Panel III report (ATP III) criteria into 47 patients with metabolic syndrome (as Group I) and 25 patients without metabolic syndrome (as Group II). Components of the ATP III criteria are the Triglycerides (TG) >150 mg/dL, (Fasting blood sugar) FBS >100 mg/dL, blood pressure (BP) >130/80, HDL‐C in women ≤50 mg/dL and in men ≤40 mg/dL, and Waist circumference in women ≥88 cm and in men ≥102 cm.^4^ The control group consisted of 40 non‐diabetic healthy people who did not fulfil the criteria for metabolic syndrome and were age‐ and sex matched with patients. All subjects voluntarily agreed to take part in the study and gave their written consent. This study was approved by the ethics committee of Golestan University of Medical Sciences (10059004136).

### 2.2 Laboratory measurements

Patients with type 2 diabetes were referred to the laboratory by the physician. BP was measured with manometer, height and waist circumference with meters and weight with a standard scale. Then, 5 mL of fasting blood samples (after 12 h of fasting) were collected in test tubes from the three groups of this study (112 subjects). Separated sera stored at −70°C until to be used. FBS, cholesterol, TG, HDL‐C and LDL‐C were measured in the sera by the enzymatic method (Pars Azmoon Kit, Iran). Serum concentrations of ANGPTL 3 and 4 were measured using commercially available Human ELISA kits (Biovendor, Czech Republic) according to the kit instructions.

### 2.3 Statistical analysis

To analyse the data, SPSS software v. 18 was applied. Differences between subjects with and without metabolic syndrome were compared by ANOVA and multiple comparison tests. For variables with abnormal distribution, Kruskal–Wallis and Mann–Whitney‐*U* tests were used. Results were shown as mean ± standard deviation (SD) and all differences with a *p*‐value of ≤0.05 mentioned as statistically significant. All assays were performed at least three times.

## 3 RESULTS

Characteristics of the subjects who were included in the study are shown in Table 1. Subjects are grouped on the basis of metabolic syndrome status. Overall, 112 serum specimens (72.3% female and 27.6% male) were examined. With regard to age and sex, no significant difference was found among the groups.

**TABLE 1:** Anthropometric characteristics of subjects.

Anthropometric characteristics	Group I (*n* = 47)	Group II (*n* = 25)	Group III (*n* = 40)	*p*‐Value
Age (years)	53.31 ± 10.40	53.76 ± 14.03	50.05 ± 11.77	.348
Body mass index (kg/m^2^)	29.96 ± 4.53	26.40 ± 9.69	26.79 ± 2.74	.000
Body weight (kg)	79.46 ± 14.82	67.98 ± 21.72	71.37 ± 9.52	.000
Waist circumference (cm)	101.34 ± 13.18	86.59 ± 1311.24	100.92 ± 15.70	.000

*Note*: *n*: number of subjects, Data are defined as means ± standard deviation (SD). Group I, Diabetic patients with metabolic syndrome; Group II, Diabetic patients without metabolic syndrome, Group III, Patients without diabetes and metabolic syndrome. *p* Value ≤.05 was considered as significant.

**FIGURE 1:**
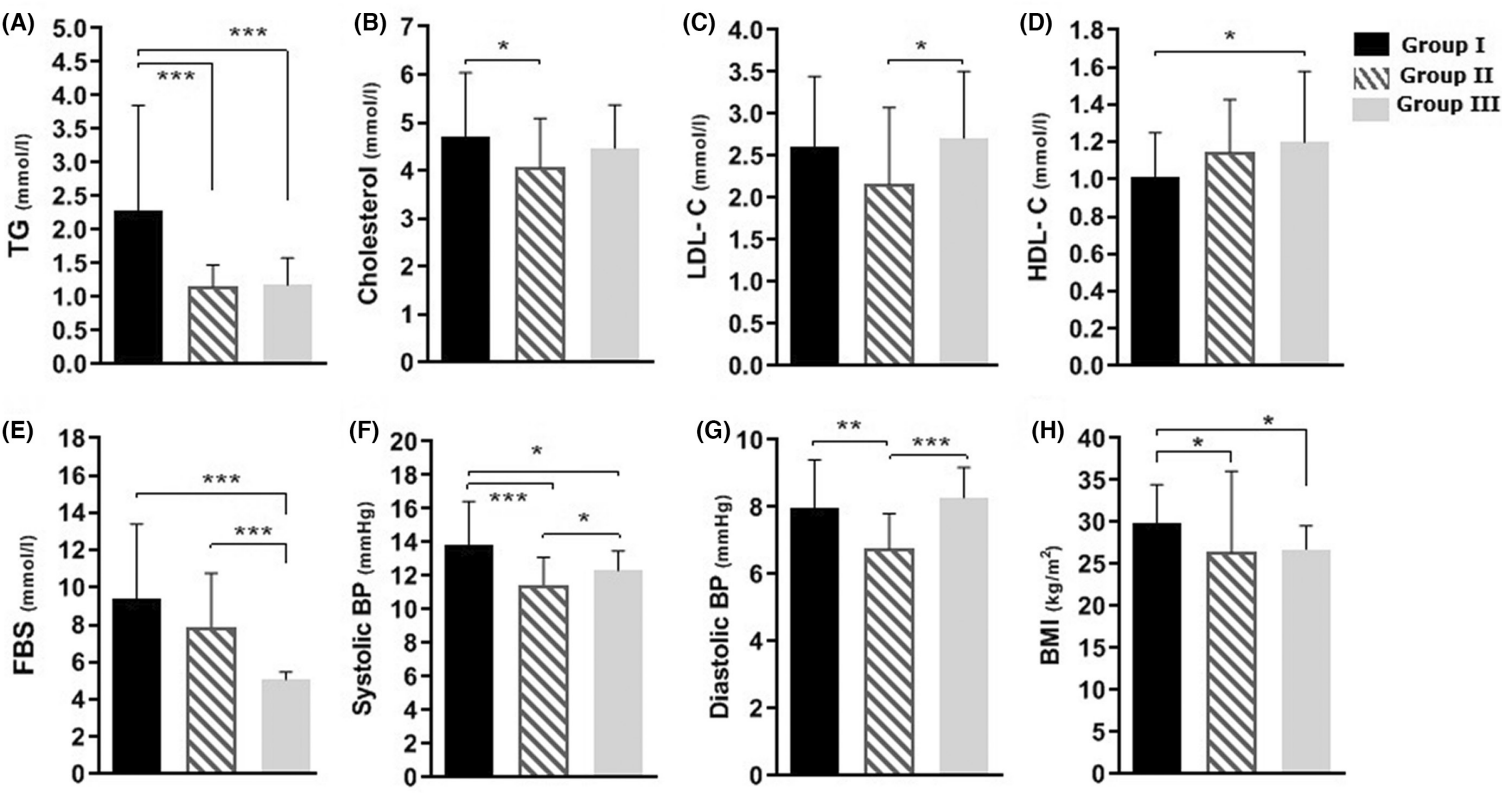
Biochemical parameters of subjects. FBS, fasting blood sugar; HDL, high density lipoprotein; LDL, low density lipoprotein; TG, triglyceride. Group I, Diabetic patients with metabolic syndrome; Group II, Diabetic patients without metabolic syndrome, Group III, Subjects without diabetes and metabolic syndrome. *p* Value ≤.05 was considered as significant. *p < .05; **p < .01; ***p < .001.

The results of the biochemical tests in the participants are presented in Figure 1. Also, Table 2 represents descriptive statistics of the serum ANGPTL 3 and 4 levels in the subjects.

**TABLE 2:** Angiopoietin‐like protein serum levels of subjects.

Variable (*p*‐Value)	Groups (mean ± SD)		*p*‐Value	Effect size (Cohen's d)
ANGPTL3 (ng/mL) (0.000)	Group I (1.46 ± 2.16)	Group II	.408	0.0404
		Group III	.000	0.4063
	Group II (1.37 ± 2.06)	Group III (4.87 ± 9.37)	.007	0.4199
ANGPTL4 (ng/mL) (0.844)	Group I (2.62 ± 4.47)	Group II	.523	0.1830
		Group III	.685	0.2412
	Group II (3.60 ± 6.11)	Group III (5.02 ± 13.34)	.961	0.1368

*Note*: Data are presented as mean ± SD. Effect size are shown base on Cohen's d (0.2: small effect, 0.5: medium effect, 0.8: large effect, 1.5: very large effect). Group I: Diabetic patients with metabolic syndrome, Group II: Diabetic patients without metabolic syndrome, Group III: Subjects without diabetes and metabolic syndrome (control group). ANGPTL, angiopoietin‐like protein. *p* Value ≤.05 was considered as significant.

As brief, A significant difference was found between patients with and without metabolic syndrome (Group I versus Group II) in the case of the systolic BP (*p* = .000), diastolic BP (*p* = .002), TG (*p* = .000), cholesterol (*p* = .025) and BMI (*p* = .032). However, no significant difference was found between these two groups for the HDL‐C (*p* = .061), LDL‐C (*p* = .057), FBS (*p* = .077), ANGPTL 3 (*p* = .408) and ANGPTL 4 (*p* = .523).

There was a significant difference between diabetic patients with metabolic syndrome (Group I) and the control group (Group III) in systolic BP (*p* = .001), FBS (*p* = .000), TG (*p* = .000), HDL‐C (*p* = .023), ANGPTL 3 (*p* = .000). But, this difference was not statistically remarkable for the diastolic BP (*p* = .084), cholesterol (*p* = .322), LDL‐C (*p* = .562), and ANGPTL 4 (*p* = .685).

In addition, there was a significant difference between diabetic patients without metabolic syndrome (Group II) and the control group (Group III) in the systolic BP (*p* = .034), diastolic BP (*p* = .000), FBS (*p* = .000), LDL‐C (*p* = .019) and ANGPTL 3 (*p* = .007); while no significant difference was indicated for the TG (*p* = .701), cholesterol (*p* = .174), HDL‐C (*p* = .772), and ANGPTL 4 (*p* = .961) (Figure 1 and Table 2).

Also, the results of the correlation coefficient between the evaluated parameters and the serum protein levels of ANGPTL4 and ANGPTL3 showed that there was a significant negative correlation between ANGPTL3 with FBS (*p* = .000), TG (*p* = .003) and BMI (*p* = .021). While this significant negative correlation was also shown between ANGPTL4 and TG (*p* = .019). Also, a significant positive correlation between HDL and ANGPTL3 was revealed (*p* = .045). On the other hand, a significant positive correlation was revealed between ANGPTL4 and ANGPTL3 (*p* = .000) (Table 3).

**TABLE 3:** Correlation coefficient between biochemical parameters of subjects.

		FBS	TG	Chol	LDL	HDL	ANGPTL3	ANGPTL4	BMI	SBP
ANGPTL3	Correlation Coefficient	−0.402^a^	−0.277^a^	−0.073	−0.080	0.190^b^		0.704^a^	−0.219^b^	−0.190
	Sig. (2‐tailed)	0.000	0.003	0.444	0.428	0.045		0.000	0.021	0.052
ANGPTL4	Correlation Coefficient	−0.142	−0.250^b^	−0.102	−0.086	0.062	0.704^a^		−0.132	−0.160
	Sig. (2‐tailed)	0.188	0.019	0.345	0.427	0.565	0.000		0.220	0.153

^a^ Correlation is significant at the 0.01 level (2‐tailed).

^b^ Correlation is significant at the 0.05 level (2‐tailed).

Abbreviations: ANGPTL, angiopoietin‐like protein; BMI, body mass index; Chol, cholesterol; FBS, fasting blood sugar; HDL‐C, high density lipoprotein‐cholesterol; LDL‐C, low density lipoprotein‐cholesterol; SBP, systolic blood pressure; TG, triglycerides.

## 4 DISCUSSION

In the present study, the serum level of ANGPTL 3 in both diabetic groups with and without metabolic syndrome (Groups I and II) was significantly lower than the control group.

Consistent with our finding, Cinkajzlova et al. showed that obese patients with and without type 2 diabetes mellitus had lower levels of ANGPTL3.^17^

ANGPTL3 has an important role in the regulation of lipoprotein metabolism through the inhibition of LPL enzyme. This enzyme hydrolyzes the apolipoprotein B‐containing lipoproteins chylomicrons and VLDL (very low‐density lipoprotein) and results in the release of TG.^19^, ^20^, ^21^ The absence of ANGPTL3 leads to an enhanced LPL activity that increases the hydrolysis of TG in TG‐rich lipoproteins.^6^, ^22^, ^23^

In human subjects with ANGPTL3 deficiency, levels of cholesterol are lower in plasma lipoprotein fractions. In these patients, TG, particularly HDL‐C and VLDL lipoproteins are reduced.^24^, ^25^ Also, mutations which impair actions of ANGPTL3 lead to the emergence of familial combined hypolipidemia.^26^, ^27^, ^28^, ^29^, ^30^

Shimamura et al. revealed that ANGPTL3 deficient mice had low levels of plasma HDL‐C. They suggested that ANGPTL3, as an inhibitor of endothelial lipase, maybe play a role in the regulation of plasma HDL‐C levels in humans and rodents.^31^

In our study, the Group I had higher levels of serum TG, cholesterol and BMI than the control group while their serum HDL‐C level was decreased. it may be concluded that our results confirm that ANGPTL3 have an important role in the regulation of serum TG, cholesterol, and HDL‐C levels by affecting LPL activity.^6^, ^31^ Therefore, the decreased levels of ANGPTL3 put the person at an increased risk for type 2 diabetes development and cardiovascular diseases.

In contrast with our findings Guo et al. showed that there was no remarkable difference between patients with metabolic syndrome and the healthy subjects in the case of serum ANGPTL 3 level.^32^

Also, our results revealed that the serum level of ANGPTL 4 in both diabetic groups with and without metabolic syndrome was lower than the control group. In addition, the level of this glycoprotein in type 2 diabetic patients with metabolic syndrome was lower than patients without metabolic syndrome. These findings were not statistically significant.

ANGPTL4 similar to ANGPTL3 inhibits LPL enzyme and through this mechanism blocks the release of non‐esterified fatty acids and their subsequent absorption.^6^

Interestingly, insulin actions result in the reduction of the plasmatic level of ANGPTL4. It has been shown that in the course of hyperinsulinemic‐euglycemic clamp in healthy individuals the plasmatic level of this glycoprotein and its mRNA in adipose tissue were decreased by insulin.^33^ It is assumed that this glycoprotein is involved in the pathogenesis of type 2 diabetes mellitus and metabolic syndrome which are associated with dyslipidemia.^21^ Animal studies in mice have demonstrated that ANGPTL4 stimulates hyperlipidemia and hepatic steatosis but reduces blood glucose and ameliorates glucose tolerance.^23^

Peroxisome proliferation activators target the gene of this glycoprotein and regulate its expression. Agonists of these activators are broadly used as the antidiabetic and lipid‐lowering agents.^23^

Xu et al. showed that ANGPTL4 directly affects regulation of lipid metabolism, glucose homeostasis, and insulin sensitivity. Expression of this glycoprotein in C57 mice reduced blood glucose and ameliorated glucose tolerance but led to the fatty liver and hyperlipidemia. In diabetic mice, ANGPTL4 treatment decreased glucose to a normal level, and significantly improved glucose intolerance. According to the results of Ex vivo experiments, this glycoprotein significantly decreases glucose production by the liver and intensifies insulin‐mediated inhibition of gluconeogenesis. Also, this group showed that levels of ANGPTL4 in the human serum have an inverse correlation with plasmatic glucose level and the homeostasis model assessment of insulin resistance. Type 2 diabetic patients had lower levels of the serum ANGPTL4 in comparison to the healthy people, suggesting that the reduced ANGPTL4 could be a risk factor for the disease.^23^

In contrast, in a study by Cinkajzlova et al. 23 patients with obesity and 40 patients with both obesity and type 2 diabetes mellitus were examined for the serum ANGPTL3 and 4. Results of the study indicated that obese patients without or with type 2 diabetes mellitus had elevated levels of the ANGPTL4 rather than the healthy control individuals.^17^

Tjeerdema et al. demonstrated that in patients with metabolic syndrome, metabolic syndrome plus inflammation, and type 2 diabetes mellitus, plasma ANGPTL4 and TG levels were higher than the healthy controls while the HDL‐C levels were significantly lower.^1^

Furthermore, Dikker et al. discovered that obese adolescents had increased levels of ANGPTL 4, TG, cholesterol, and BMI levels.^34^ Therefore, they couldn't find a relationship between ANGPTL 4 and hepatosteatosis.

On the basis of our results for the ANGPTL4, FBS, TG, cholesterol, HDL‐C and BMI, it maybe concluded that ANGPTL4 modulates TG, cholesterol and HDL‐C levels by the inhibition of LPL activity. Therefore, a reduction in the serum level of this glycoprotein probably could be considered as a risk factor for type 2 diabetes.

## 5 CONCLUSION

Overall, our findings suggest that the decreased levels of ANGPTL 3 and 4 could be a causative factor for type 2 diabetes.

## AUTHOR CONTRIBUTIONS

**Sharabeh Hezarkhani:** Conceptualization (equal); validation (equal). **Aytekin Hajighaderi:** Methodology (equal). **Sara Hosseinzadeh:** Writing – original draft (equal). **Naser Behnampour:** Formal analysis (equal); software (equal). **Gholamreza Veghari:** Supervision (equal). **Farshid Fathabadi:** Writing – review and editing (equal). **Zahra Hesari:** Writing – original draft (equal); writing – review and editing (equal). **Hamid Reza Joshaghani:** Project administration (equal); validation (equal).

## FUNDING INFORMATION

The financial support of this study was provided by the grant number 9003170124 from Golestan University of Medical Sciences.

## CONFLICT OF INTEREST STATEMENT

The authors declare that they have no conflict of interest.

## ETHICS STATEMENT

Ethical Approval for this research study was accepted by Golestan University of Medical Sciences Ethics Committee (10059004136).

## CONSENT TO PARTICIPATE

During collecting data, the researchers obtained informed consent from the participants after explaining the purpose and objective of the study.

## Data Availability

The data that support the findings of this study are available from the corresponding author upon reasonable request.
